# Glycine Supplementation Ameliorates Retinal Neuronal Damage in an Experimental Model of Diabetes in Rats: A Light and Electron Microscopic Study

**DOI:** 10.18502/jovr.v14i4.5449

**Published:** 2019-10-24

**Authors:** Soghra Gholami, Younes Kamali, Mohammad Reza Rostamzad

**Affiliations:** ^1^Department of Basic Sciences, School of Veterinary Medicine, Shiraz University, Shiraz, Iran; ^2^Department of Basic Sciences, School of Veterinary Medicine, Shiraz University, International Division, Shiraz, Iran

**Keywords:** Diabetic Retinopathy, Electron Microscopy, Experimental, Glycine, Light Microscopy, Rat

## Abstract

**Purpose:**

To investigate the potential neuroprotective effect of glycine supplementation on the retinal ultrastructure of streptozocin (STZ)-induced diabetic rats.

**Methods:**

Adult male Wistar rats weighing 200–250 g (*n* = 40) were randomly divided into four groups of 10 each: normal group (C), glycine + normal group (G), STZ group (D), and glycine + STZ group (DG). The G and DG groups received glycine (130 mM and 1% w/v) freely in their drinking water seven days after the induction of diabetes for up to 16 weeks. Retinal samples for histopathology were examined using light and electron microscopy.

**Results:**

Diabetes-induced histological changes were attenuated in the retinas of rats in the DG group. The ultrastructural alterations produced by experimental diabetes in the inner nuclear layer, outer nuclear layer, and ganglion cell layer were significantly ameliorated by glycine supplementation.

**Conclusion:**

Our findings suggest that glycine supplementation effectively attenuates retinal neuronal damage in experimental diabetic rats, and thus may be a potential candidate to protect retinal ultrastructure against diabetes.

##  INTRODUCTION

Diabetic retinopathy (DR) is a progressive disease which involves more pathology than solely vascular lesions as was previously believed. The impairments of various neurons of the retina, namely photoreceptor cells and ganglion cells, begin early in DR, prior to vascular damage.^[1,2,3]^ Most previous research has shown that neuronal changes of the retina result secondary to microangiopathy, but a growing body of evidence suggests that neuroretinal abnormalities may develop independently of microvascular insult.^[4,5]^

To prevent the development of retinopathy, glycemic control should be initiated in the early stages of diabetes. In most patients with non-insulin-dependent diabetes, however, retinopathy remains the leading cause of vision loss due to delayed detection and uncontrolled hyperglycemia.^[6]^ Given that insulin has a key role in the metabolism of amino acids and proteins, amino acid deficiency due to reduced insulin levels could be a significant factor in the etiology of DR^[7,8]^. Free amino acids play a crucial role in the prevention of diabetic complications, including DR, in several ways: glycemic regulation, competition with tissue proteins for non-enzymatic glycation, and inhibition or reduction of oxidative stress.^[9,10]^ The nonessential amino acid glycine is involved in a broad spectrum of biochemical processes related to cytoprotection via binding to its different receptors.^[11]^ There exist a number of *in-vivo* preclinical and clinical studies regarding the antidiabetic effects of dietary glycine supplementation.^[12]^ There is strong evidence that vessels and neurons of the retina undergo inflammation involving both innate and adaptive immunity in the pathogenesis of DR.^[13]^ Glycine administration modulates immune responses, protecting tissues from injury induced by active proinflammatory cytokines in subjects with type 2 diabetes.^[14]^ In experimentally induced diabetic rats, the vascular complications of diabetes have been ameliorated with glycine treatment.^[15]^ It is thought that glycine acts via the regulation of hyperglycemia, hypercholesterolemia, and glycated hemoglobin (A1C) levels.^[16]^ In two other studies, the effects of glycine on the prevention of cataract progression have been demonstrated.^[17,18]^

Our study was designed to evaluate histologically whether glycine supplementation in the drinking water of streptozocin (STZ)-induced diabetic rats has a protective effect on the retinal ultrastructure, with a focus on the ganglion and photoreceptor cell layers.

##  METHODS

###  Animals and Induction of Diabetes

Adult male Wistar rats weighing 200–250 g (*n* = 40) were obtained from the Razi Vaccine and Serum Research Institute of Shiraz. The animals were divided into four groups of 10 each: normal (control) group (C), glycine + normal group (G), STZ group (D), and glycine + STZ group (DG). Groups C and G were intraperitoneally injected with physiological saline and served as normal group. Groups D and DG were administered intraperitoneally with a single 65 mg/kg dose of STZ (Sigma-Aldrich, USA). Rats were considered diabetic and used in the study if fasting blood glucose concentration exceeded 240 mg/dl, 24 h and 7 days after the STZ injection. The second (G) and fourth (DG) groups received glycine at a concentration of 130 mM and 1% w/v freely in their drinking water from day 7 onward up to 16 weeks. Body weight (g) and blood glucose level were measured and recorded at the time of sacrifice 16 weeks after the onset of diabetes for each group of rats.

All experiments were carried out according to the National Institute of Health Guidelines for the care and use of laboratory animals and the European Council Directive on November 24, 1986 for the Care and Use of Laboratory Animals (86/609/EEC) and approved by the Local Ethics Committee.

###  Histological and Electron Microscopic Examination

At the end of the 16-week period, animals were lethally injected with a combination of ketamine and xylazine; after cervical dislocation, the eyeballs were removed immediately and the anterior portions, including the cornea, lens, and vitreous body were carefully excised. The posterior eyecups were fixed in 2.5% glutaraldehyde in 0.1 M sodium phosphate buffer (pH 7.3) for 3 h at 4°C. After fixation, the retinas were carefully dissected from the choroids, and small pieces were taken from the central region near the optic disc. The tissues were then repeatedly rinsed with 0.1 M sodium cacodylate buffer (CB) at room temperature, and routine osmication was carried out with 1% osmium tetroxide in 0.1 M CB for 1 h at 48°C. After repeated washing with distilled water, tissues were dehydrated with a graded series of alcohol. Samples were then placed in a mixture of propylene oxide and TAAB resin and embedded in pure resin. Semi-thin and ultra-thin sections were cut using an OMU3 ultramicrotome C. Reichert (Austria). For each rat, three to five semi-thin sections were stained with toluidine blue for preliminary histological examination. A qualitative evaluation based on a simple grading system (absent (0), mild (1), moderate (2), severe (3)) was used for all specific histopathological features. For transmission electron microscopy (Philips CM10), ultra-thin sections (70-nm thick) were mounted on 200 mesh uncoated copper grids and stained with uranyl acetate followed by lead citrate.

###  Statistical Analysis

Data were presented as the mean ± standard deviation. A one-way ANOVA with Tukey's test was used for the statistical analyses of body weight and blood glucose between the different experimental groups. The Mann–Whitney nonparametric test was used to compare histological scores between the D and DG groups. *P* < 0.05 was considered statistically significant.

##  RESULTS

###  Body Weight and Blood Glucose Levels

As shown in Table 1, average body weight was significantly lower in the D and DG groups 16 weeks after the induction of diabetes compared to the C and G groups (*P* < 0.05). The diabetic rats gained less weight from day 0 to 16 weeks compared to the healthy animals (4% vs. 70%). Rats in the glycine-treated diabetic group exhibited a 9% weight loss. Serum glucose levels were significantly higher in the diabetic rats compared to the healthy animals on both days tested (*P* < 0.05). At the end of the experiment, the blood glucose levels of the glycine-treated diabetic rats were less than that of the diabetic rats, though this difference was not statistically significant (398.17 ± 38.23 compared to 455.53 ± 41.14, *P* < 0.05).

**Table 1:** Body weight and serum glucose levels in the experimental groups (Day 0 = Day of diabetes induction).


	**Body Weight ± SD (g)**		**Blood Glucose ± SD (mg/dl)**	
**Group**	**Day 0**	**Week 16**	**Day 0**	**Week 16**
C	218.43${}^{a}$ ± 30.12	369.68${}^{a}$ ± 54.17	80.67${}^{a}$ ± 4.19	83.21${}^{a}$ ± 3.38
G	224.81${}^{a}$ ± 18.63	342.83${}^{a}$ ± 46.17	93.22${}^{a}$ ± 3.29	93.45${}^{a}$ ± 4.29
D	202.18${}^{a}$ ± 22.15	210.23${}^{b}$ ± 16.39	438.45${}^{b}$ ± 48.43	455.53${}^{b}$ ± 41.14
DG	235.01${}^{a}$ ± 27.68	214.75${}^{b}$ ± 20.44	441.25${}^{b}$ ± 58.22	398.17${}^{b}$ ± 38.23
	
	
${}^{a,b}$At each column, different superscript alphabets show a significant difference				
(*P* < 0.05); all data are presented as mean ± SD				

###  Light Microscopy

**Figure 1:**
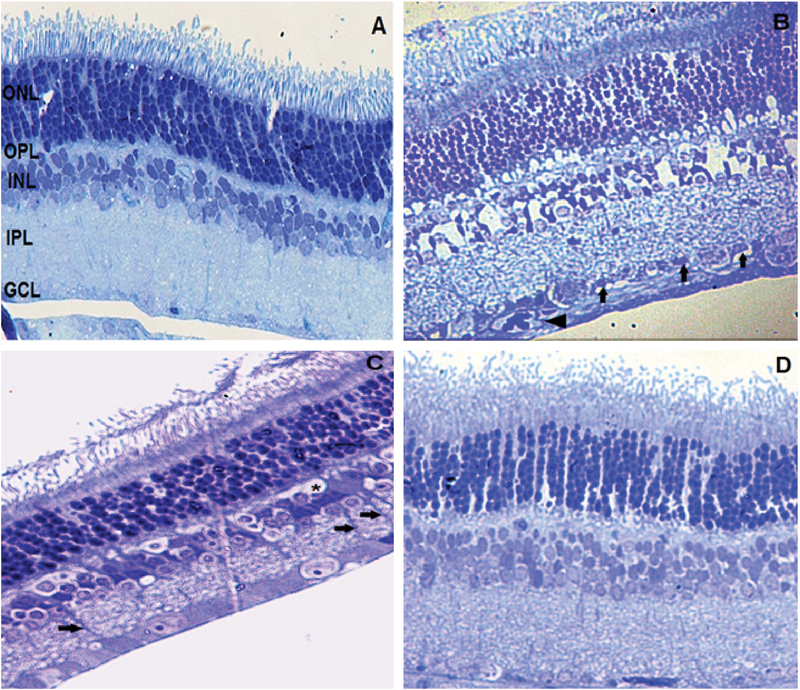
(A) The retinal architecture in groups C and G is organized into regular layers. (B) & (C) The retina in group D showed remarkable cell death in the ONL and INL, severe vacuolation of the IPL and spongiform OPL, heavily stained, shrunken nuclei in the GCL, and hypertrophy of the radial processes of Müller cells (arrows). Arrowhead: A blood vessel projecting via the GCL into the nerve fiber layer; Asterisk: Scattered anucleate ghost cells within the INL. (D) the structure of retinal layers in group DG was more regular and normal. (×400 magnification) ONL, outer nuclear layer; INL, inner nuclear layer; IPL, inner plexiform layer; OPL, outer plaxiform layer; GCL, ganglion cell layer.

Light microscopy assessment of the retinal sections of the C and G groups revealed no recognizable differences in the histological profile [Figure 1(A)]. The retina of rats in group D showed diverse histological lesions. The photoreceptor cells showed severe degeneration and exhibited hollow spaces between them. In the inner nuclear layer (INL), most cells displayed condensation of nuclear chromatin and cytoplasmic vacuolization, whereas others were ghost cells due to nuclear disintegration. In most sections, the appearance of the inner plexiform layer (IPL) and outer plexiform layer (OPL) was vacuolated and spongy. An increased number of shrunken cells with dark nuclei was present in the ganglion cell layer (GCL), reflecting cellular degeneration [Figure 1(B)]. Hypertrophy of Müller glial processes in response to damage was observed, and the vitreal radial processes within the GCL and IPL and the scleral radial processes between the columns of photoreceptor cells stained plainly [Figure 1(C)]. In contrast to group D, only slight histological changes were seen in the glycine-treated diabetic (DG) group. Most photoreceptor cells and their cellular attachments were preserved. The empty spaces within the INL were nearly absent. The IPL and OPL also showed a uniform reticular appearance with narrow spaces between its fibers. Cells in the GCL were also nearly all intact in appearance [Figure 1(D)]. Detailed histopathologic results are shown in Table 2. With respect to the various histological features noted in Table 2, eight rats in the DG group exhibited no change in retinal sections or mild to moderate lesions.

**Table 2:** Histological scoring of retinal tissues in the D and DG groups.


**Groups**	**CD in ONL**	**Vacuolation of IPL**	**CD in INL**	**Vacuolation of OPL**	**Shrunken nuclei in GCL**	**Hypertrophy of MGPs**	**Disorganization of RLs**
D	M ± SD	2.7 ± 0.48*${}^{*}$	2.4 ± 0.52*${}^{*}$	2.9 ± 0.32*${}^{*}$	2.6 ± 0.70*${}^{*}$	2.7 ± 0.48*${}^{*}$	2.1 ± 0.87	2.7 ± 0.49*${}^{*}$
	Median	3	2	3	3	3	2	3
DG	M ± SD	1.9 ± 0.74	1.6 ± 0.70	1.7 ± 0.48	1.6 ± 0.70	1.7 ± 0.48	1.8 ± 0.79	1.3 ± 0.95
	Median	2	2	2	1.5	2	2	1
	
	
CD, cellular degeneration; ONL, outer nuclear layer; IPL, inner plexiform layer; INL, inner nuclear layer; OPL, outer plexiform layer; GCL, ganglion cell layer; MGPs, Müller glia processes; RLs, retinal layers; M ± SD, mean ± standard deviation								
Asterisk (*) at each column represents a significant difference (*P* < 0.05);								

###  Transmission Electron Microscopy

**Figure 2:**
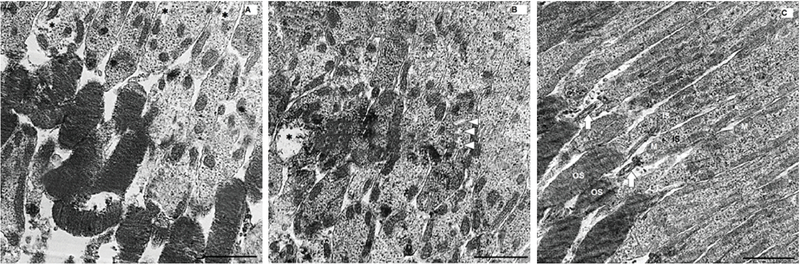
Electron micrographs showing the outer segments (OS) and inner segments (IS) in group D (A and B) and group DG (C). (A) & (B): Most of the inner segments are swollen and markedly vacuolated (asterisks), contain degenerated mitochondria (white arrowheads), and the outer segments are severely disorganized. (C) Orderly array in most of the photoreceptor segments with mild injuries. White arrows: Two connecting cilia; M, mitochondria (Scale bar, 2 µm).

**Figure 3:**
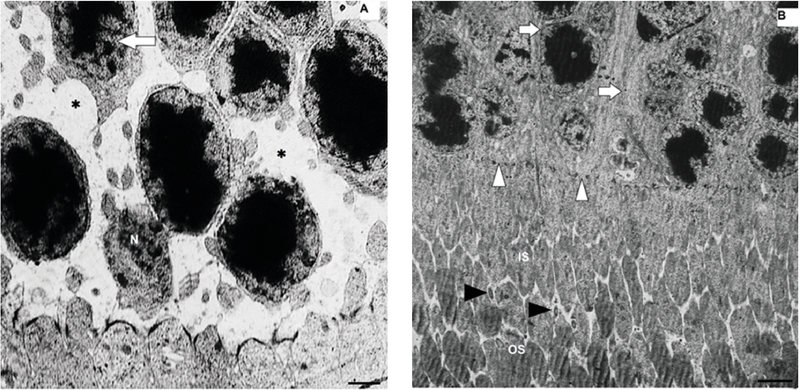
Electron micrographs of the outer nuclear layer (ONL) in group D (A) and group DG (B). (A) Enlarged intercellular spaces (asterisks); nuclear degeneration (N); cytoplasmic and nuclear vacuolization (arrow). (B) Most of the cells showed normal ultrastructure with normal cellular attachments (arrows) forming the intact external limiting membrane (white arrowheads); two connecting cilia (black arrowheads); inner segment (IS); outer segment (OS) (Scale bar is 1 µm in A, 2 µm in B).

**Figure 4:**
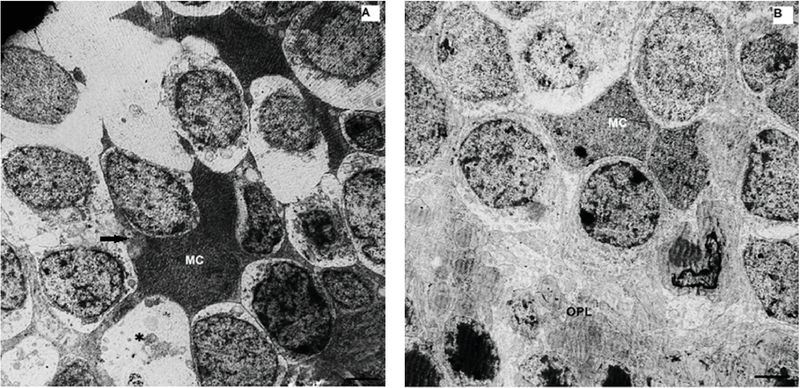
Electron micrographs of the inner nuclear layer (INL) in group D (A) and group DG (B). (A) An apoptotic Müller cell (MC) with highly condensed chromatin, membrane swelling, ruptured mitochondria (arrow), and numerous empty spaces (asterisk) containing membranous debris due to cell death. (B) Well-preserved microvilli of Müller cells (MC); OPL, outer plexiform layer (Scale bar, 2 µm).

**Figure 5:**
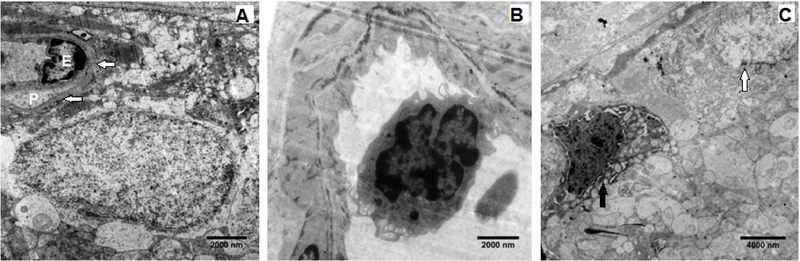
Electron micrographs of a retinal ganglion cell (RGC) in groups C and G (A), group D (B), and group DG (C). (A) An RGC with uniformly distributed chromatin throughout its nucleus. Note a typical normal retinal capillary composed of an endothelial cell (E) and pericyte (P) surrounded by the basement membrane (arrows). (B) An apoptotic RGC with shrunken nucleus containing condensed and marginated chromatin. (C) An RGC with relatively uniformly dispersed chromatin (black arrow) and a normal RGC (white arrow).

Electron microscopic analysis of the retina of diabetic rats added to the results of light microscopy. Notable derangement of the outer and inner segments, disorganized and disoriented membranous discs in the outer segment, and vacuolar swelling of the inner segment containing swollen mitochondria with lysis of their cristae (cristolysis) were the observed ultrastructural findings in photoreceptor segments [Figure 2]. Most photoreceptor cell bodies displayed cytoplasmic and nuclear vacuolization [Figure 3]. Various cells of the INL, including Müller cells, showed typical signs of apoptosis, characterized by chromatin condensation, membrane swelling, and ruptured mitochondria [Figure 4]. Significant cell death in both the ONL and INL left empty spaces containing membranous debris between the cells [Figures 3 and 4]. The presence of indented nuclei with marginal condensation in the

shrunken cells of the GCL confirmed apoptosis [Figure 5]. All of these aforementioned changes were partially attenuated by treating diabetic animals with glycine.

##  DISCUSSION

Given that the adverse effects of long-term use of antidiabetic drugs are unavoidable, research into the use of amino acids as a preferred alternative to prevent complications of diabetes may be noteworthy. The beneficial effects of amino acids on the regulation of insulin secretion and glucose levels have been documented in both preclinical and clinical studies.^[19]^ Of the free amino acids, glycine may be a suitable candidate for the prevention of DR as a result of its high antioxidant and anti-glycation properties.^[15,16]^ Although the ultrastructural changes occurring in retinopathy in experimentally diabetic rats have been previously studied, most of these have focused on microangiopathic features.^[20,21]^

Ganglion and Müller cells are the two different cell types of the retina that primarily undergo apoptosis in DR.^[22,23]^ As the main glial cells of the vertebrate retina, Müller cells play both structural and metabolic roles in supporting retinal neurons.^[24]^ It is assumed that the reactivation of Müller cells (gliosis) in response to retinopathies can cause the apoptotic death of neurons.^[25]^ Some of the key pathways involved in the progression of Müller cells gliosis include accumulation of advanced glycation end products (AGEs), inflammatory mediators, the polyol pathway, and oxidative stress.^[26,27]^ The pathological characteristics of reactivated Müller cells in DR include upregulation of the immunoreactivity of intermediate filament proteins and glial fibrillary acidic protein (GFAP), cellular hypertrophy, extension of cytoplasmic processes, and migration and proliferation.^[28]^ The processes of Müller cells in the histological retinal sections of STZ-induced diabetic rats are hypertrophic, spanning throughout the retinal thickness.^[5,26]^ This histological feature, which indicates that Müller cells dramatically reacted in response to neuronal degeneration, was attenuated in the diabetic group receiving glycine. This preventive effect of glycine against the reactivation of Müller cells can be partly attributed to its antioxidant, anti-inflammatory, and anti-glycation properties.

In our study, marked ultrastructural changes were detected in the photoreceptor layer of diabetic rats, manifested by the disorganized arrangement of cone and rod outer segments, whereas glycine supplementation greatly ameliorated these changes. Mature photoreceptor outer segments are capable of renewing themselves by adding new discs to the basal end and removing old discs from the apical end.^[29]^ In some retinal degenerative disorders such as DR, the balance between the addition and removal of membranous discs is disrupted, leading to photoreceptor degeneration.^[30]^ The cellular and molecular mechanisms by which photoreceptor degeneration develops in diabetes are largely unknown. Evidence has suggested that the development and survival of the photoreceptor cell outer segments is directly affected by hyperglycemia and hypoinsulinemia, with perturbations of insulin signaling playing a diminutive role.^[31,32]^ Park et al showed that photoreceptors begin to undergo apoptotic cell loss about four weeks after the induction of experimental diabetes and continue to progressively degenerate from week 12 onward.^[5]^ Our histological observations of apoptotic death of photoreceptors at week 16 of diabetes are in agreement with these findings. It has been reported that insulin therapy is one of the most effective therapeutic approaches in reversing the neuronal apoptosis seen in the diabetic retina.^[33]^ In humans, one study detected an elevated insulin response to the oral ingestion of glycine, even in the absence of glucose.^[34]^ Oral glycine stimulates the secretion of a gut hormone to reinforce the effect of insulin on glucose elimination from the circulation.^[34]^ The observed improved histological profile of photoreceptors in the glycine-treated diabetic (DG) group compared to the diabetic group may, therefore, be at least in part, explained by the insulin-enhancing properties of glycine supplementation.

Extensive research into the experimental DR has clearly demonstrated that although the generation of reactive oxygen species (ROS) is increased during the early stages of hyperglycemic insult, structural damage to mitochondria is only observed when hyperglycemia continues for a long period of time.^[35]^ Indeed, hyperglycemia-induced mitochondrial overproduction of ROS involves several metabolic pathways and leads to progressive injury to mitochondria, further increasing oxidative stress.^[2,36]^ Morphological alterations in mitochondria have been associated with cell death and apoptosis. In the current study, ultrastructure of mitochondria in the inner segment of photoreceptor cells was affected in the diabetic retina, suggesting a role in the apoptotic phenomenon. Glycine was able to partially prevent these changes, likely as a result of its antioxidant properties.

Research in diabetic rodent models has demonstrated that a loss of retinal ganglion cells (RGCs) occurs in the early stages of diabetes, prior to the development of visible microangiopathies. Martin et al reported that induction of experimental diabetes led to a marked increase in neuronal loss in the GCL of mice retinas via an apoptotic pathway 14 weeks later.^[37]^ In other experimental studies in rats, RGCs undergoing apoptosis were detected as early as 4 and 12 weeks after diabetes induction.^[38,39,40,41]^ Consistent with these findings, our ultrastructural examination at week 16 of diabetes demonstrated GCL apoptosis in the retinas of STZ-induced diabetic rats. When rats were treated with glycine, there was an improvement in the chromatin distribution and nuclear profiles of the cells in the GCL, and cells containing heterochromatin were rarely observed.

In conclusion, glycine supplementation effectively attenuates retinal neuronal damage in experimental diabetic rats and may therefore be a potential candidate to protect retinal ultrastructure against diabetes. To confirm this, further studies need to be conducted into the neuroprotective effects of glycine on the diabetic retina at multiple time points from the onset of diabetes. These should investigate changes in the expression and distribution of proteins typical of gliosis, for example, GFAP, in retinal glial components using western blotting and immunohistochemical studies.

##  Financial Support and Sponsorship

This research was financially supported by the grants of Shiraz University Research Council.

##  Conflicts of Interest

There are no conflicts of interest.
